# Grasping Force Control for a Robotic Hand by Slip Detection Using Developed Micro Laser Doppler Velocimeter

**DOI:** 10.3390/s18020326

**Published:** 2018-01-23

**Authors:** Nobutomo Morita, Hirofumi Nogami, Eiji Higurashi, Renshi Sawada

**Affiliations:** 1 Advanced Manufacturing Research Institute, National Institute of Advanced Industrial Science and Technology (AIST), 807-1 Shuku-machi, Tosu, Saga 841-0052, Japan; 2 Department of Mechanical Engineering, Faculty of Engineering, Kyushu University, 744 Motooka, Nishi-ku, Fukuoka 819-0395, Japan; nogami@mech.kyushu-u.ac.jp (H.N.); sawada@mech.kyushu-u.ac.jp (R.S.); 3 Department of Precision Engineering, School of Engineering, The University of Tokyo, 7-3-1 Hongo, Bunkyo-ku, Tokyo 113-8656, Japan; eiji@su.t.u-tokyo.ac.jp; 4 Research Center for Ubiquitous MEMS and Micro Engineering (UMEMSME), National Institute of Advanced Industrial Science and Technology (AIST), 1-2-1 Namiki, Tsukuba, Ibaraki 305-8564, Japan

**Keywords:** laser Doppler velocimetry, micro laser Doppler velocimeter, laser surface velocimeter, optical MEMS, force control of grasp, dexterous robotic manipulation, slip detection, tactile sensor

## Abstract

The purpose of this paper is to show the feasibility of grasping force control by feeding back signals of the developed micro-laser Doppler velocimeter (μ-LDV) and by discriminating whether a grasped object is slipping or not. LDV is well known as a high response surface velocity sensor which can measure various surfaces—such as metal, paper, film, and so on—thus suggesting the potential application of LDV as a slip sensor for grasping various objects. However, the use of LDV as a slip sensor has not yet been reported because the size of LDVs is too large to be installed on a robotic fingertip. We have solved the size problem and enabled the performance of a feasibility test with a few-millimeter-scale LDV referred to as micro-LDV (μ-LDV) by modifying the design which was adopted from MEMS (microelectromechanical systems) fabrication process. In this paper, by applying our developed μ-LDV as a slip sensor, we have successfully demonstrated grasping force control with three target objects—aluminum block, wood block, and white acrylic block—considering that various objects made of these materials can be found in homes and factories, without grasping force feedback. We provide proofs that LDV is a new promising candidate slip sensor for grasping force control to execute target grasping.

## 1. Introduction

**Figure 1:**
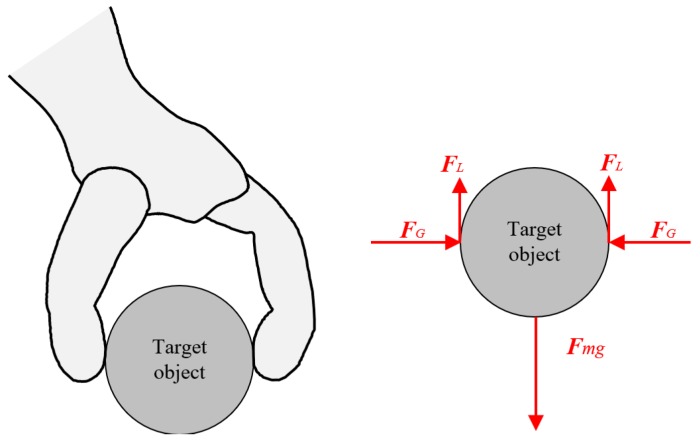
Schematic of grasping model based on Coulomb’s friction.

Robotic hands are desired in a variety of environments—such as industrial factories, hospitals, homes, etc.—for grasping and manipulation of various objects by hands as humans do. The demand is increasing year by year due to social requirements, including improvement of quality and cost performance of products, supporting medical staff and patients in hospitals, and doing housework in such places. There are various object, weight, and surface friction conditions which are unknown, but robots need to manipulate without any trouble including slippage or break of contact, damage, and destruction. We consider a simple grasping model [1] based on Coulomb’s friction model as schematized in Figure 1. Two fingers grasping a target with the grasping force FG, the gravitational force Fmg, and the lifting force FL resulting from friction are also on the target object. Here, lifting force which is described as F_L_ = μF_G_, 2F_L_ = 2μF_G_ = F_mg_ must be satisfied considering the balance of forces, where μ is static friction coefficient. However, mass and friction coefficients of household objects are usually unknown and variable. For example, the mass of a cup is changed by pouring/drinking water, and friction coefficient changes when the surface gets wet or dry. 

Therefore, direct measurement of a slip is important for adapting grasping force. Many researchers have developed slip sensors based on the principle of detecting physical changes caused by slip as follows:

(1) Slip displacement: This method measures slip directly as a displacement by rotation of a roller contacted on a target surface [2]. Slip distance, useful information to re-grasp a target object, is also detected by this method. However, the very small and high-resolution encoder with smooth shaft for avoiding misdetection results in a slip between the roller and surface of the target object. Also, mechanical play of a roller or its fixture may delay response time. 

(2) Deformation of soft robotic finger surface like skin [1,3]: The sensors estimate slip based on the deformation of the finger surface by vision sensor such as CCD camera with image processing. However, the size of the vision sensor is too large to be embedded into the robotic finger, and high computational cost is required for vision processing.

(3) Thermal flux change (thermal flux sensing) [4,5]: This type of sensor can be mounted on the curvature surface, and has the advantage of low cost, since this sensor consists of simple components such as thin polymer bendable film, electrode, and microheater that are fabricated by photolithographic process. When slip occurs, thermal flux near the microheater changes since the contact point of microheater and target surface changes, resulting in resistance of the microheater due to temperature change. However, considering the situation of grasping a rough surface object, there may be an air gap between the microheater and the surface of the target object, resulting in slipping, the sensor being insensitive, or response time becoming longer, since thermal conductivity of air is generally much lower than solid objects.

(4) Pressure sensitive materials: This method is based on the detection of rapid electrical property change of pressure sensitive materials induced by pressure distribution change due to slip. The following pressure sensitive materials are used: a pressure-sensitive rubber [6,7,8,9], a piezoresistive material [10,11], piezoelectric materials such as PZT (lead zirconate titanate) [12] and PVDF (polyvinylidene fluoride) [13,14,15]. This method has milli-seconds-order high response speed, thus being superior in detecting incipient slip. However, the performance of microvibration-based method and electrical characteristic change-based method depends on surface finishing [1]. Extensive descriptions of each slip sensing method are discussed in the review articles written by Francomano et al. [1] and Zhanat et al. [16]. 

As mentioned above, each slip sensor has its drawbacks in some cases in terms of sensitivity, response speed, or size. Thus, there is still no absolute method which can detect slip for any object in homes, hospitals, and factories with high response speed for quick manipulation similar to a human, with an acceptable sensor size.

Therefore, we propose another solution to detect slip through the use of a laser Doppler velocimeter (LDV). LDV is well known as a high response speed velocity measurement technique, which can measure various surfaces—such as metals, papers, films etc.—by irradiating a laser beam and detecting the laser scatter by the measurement target [17,18]. LDV also has wavelength-order (i.e., 1.6 µm) high displacement sensitivity that is advantageous in detecting incipient slip. However, the size of an LDV is large for a robotic finger, due to its constituent elements and requirement of assembling accuracy as described later in the Section 2.2. This limits the application of LDV as a slip sensor for a robotic hand. We have developed a micro laser Doppler velocimeter (µ-LDV) by modifying the design adopted from MEMS (microelectromechanical systems) fabrication process [19,20]. The size of the µ-LDV chip (2.8 × 2.8 × 1 mm) is small enough to be installed on a robotic fingertip. 

The purpose of this paper is to show the feasibility of grasping force control by feeding back LDV-based slip sensing results, without grasping force feedback. By applying the µ-LDV as a slip sensor, we have successfully demonstrated grasping force control with three target objects—aluminum block, wood block, and white acrylic block—as various material samples existing in homes and factories, without grasping force feedback. We provide proofs that laser Doppler velocimetry could be a new candidate slip sensor for grasping force control of unknown target objects. We have discussed other signal processing and re-designing that have potentials in increasing its performance in terms of higher response time and sensitivity. We also discussed the potential drawbacks of LDV type slip sensor and measures. 

## 2. Materials and Methods 

### 2.1. Measurement Principle of Laser Doppler Velocimeter

**Figure 2:**
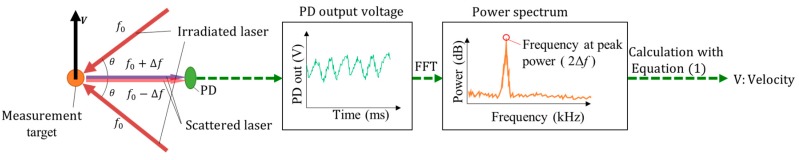
Schematic of the principle of laser Doppler velocimeter and signal processing.

LDV is based on the principle of Doppler shift; the frequency of lights shift when it is scattered by a moving object. The frequency of lights is hundreds-THz-order, which is too high frequency to be detected directly with photodiode (PD). Thus, LDV measures Doppler shifted frequency by interferometory as shown in Figure 2. Two laser beams from a laser source are irradiated onto the measurement target. Two laser beams are scattered, and one of the laser beams is Doppler shifted from the frequency f_0_ to f_0_ + Δf, beside the other laser beam Doppler shifted from the frequency f_0_ to f_0_ − Δf. These lasers interfere on PD, then the laser intensity fluctuates with the frequency difference of these lasers 2Δf, which is expressed as

(1) $$V=\frac{2∆f\lambda }{2\sin\theta }$$

where, λ, θ, and V are wavelengths of the laser, the half of intersecting angle of two laser beams, and velocity of measurement target, respectively. For further details of the measurement principle and its calculation, refer to [18]. Signal processing time of FFT is fast (i.e., sub-millisecond). Thus, the response time of LDV strongly depends on the required velocity resolution, which is inversely proportional to data acquisition time for an FFT calculation, which is arbitrarily changeable from the side of data acquisition system. The shorter data acquisition time allows for higher response times at the expense of velocity resolution and displacement resolution, involving the risk of misdetection of slow slip. Therefore, FFT setting is an important parameter for slip detection. 

### 2.2. Design of LDV and µ-LDV

**Figure 3:**
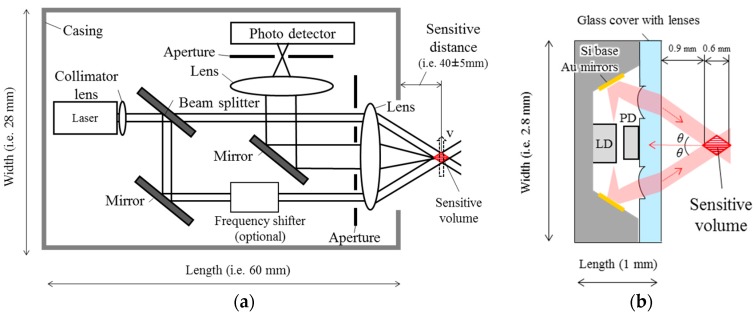
Schematic of an LDV: (**a**) The structure and components of basic LDV, displayed example of the length (60 mm), and sensitive distance (40 ± 5 mm) produced one of the smallest commercialized LDV (MODEL 1192, Act Electronics Corp., Kawasaki, Japan). (**b**) The structure and components of the developed µ-LDV.

An LDV consists of many optical components; a laser source, a beam splitter, mirrors, lenses, a photo detector, and apertures (Figure 3a). The total size should be at least over several centimeters because the size of each component is from millimeter-order to centimeter-order. Moreover, these optical components need to be aligned with high accuracy from micrometers to tens of micrometers. Hence, manipulation space for active alignment is also needed, thus requiring a larger total size. Even, the smallest commercialized LDV (MODEL 1192, Act Electronics Corp., Kawasaki, Japan) is 28 × 18 × 60 mm^3^, which is still big for a robotic finger. We overcome the difficulty of miniaturization of LDV with an innovative design concept: miniaturizing each component, reducing number of parts by monolithic integration of each component at high machining accuracy by adopting MEMS, (microelectromechanical systems) fabrication process, and omitting a beam splitter and apertures. The size (2.8 × 2.8 × 1 mm-thick) is small enough to be installed on a robotic fingertip. A section schematic of the developed µ-LDV is shown in Figure 3b. The µ-LDV has a bare chip photodiode (PD), and a bare chip laser diode (LD) (1310 nm-wavelength and 5 mW-power). By using bare chips, instead of packaged diodes, both the diode size and distance between diodes and other components can be significantly reduced. We used double-sided emitting LD, enabling omission of a beam splitter. Apertures and a lens for collecting scattering light are also omitted to reduce parts, although this change may signal lower intensity and signal-noise ratio. The mirrors, Si base, lenses, and glass cover are monolithically fabricated to reduce the total size, and alignment process of each component is unnecessary. The µ-LDV irradiates two laser beams (beam diameter: φ 0.3 mm), then laser beams cross at 1.2 mm away from the sensor surface. The sensitive distance is from 0.9 mm to 1.5 mm from the surface of the µ-LDV. The relation between velocity of moving target V, and the beat frequency f_beat_ (=2Δf) is f_beat_ = 0.615 V, is as calculated from Equation (1), where half of crossing angle of laser beams θ is 22.3°. In other words, PD output signals oscillate one cycle per 1.62 µm displacement of measurement target, which means that the µ-LDV potentially has high sensitivity to slip displacement.

**Figure 4:**
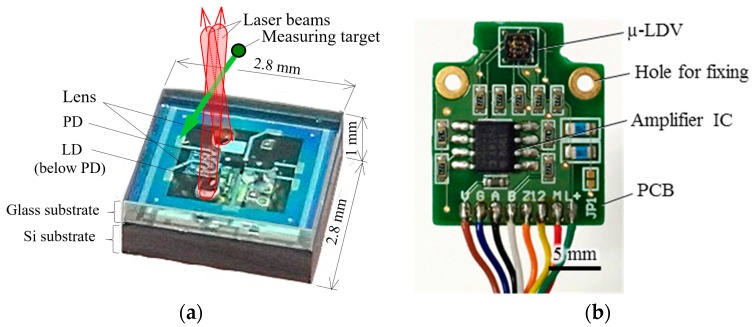
The developed slip sensor called µ-LDV: (**a**) A photo of the µ-LDV with a schematic of laser beam irradiation. The cross volume of laser beams is measurable volume of slip velocity; (**b**) A photo of the µ-LDV installed onto PCB with fixing through-hole, wiring, and pre-amplifier circuit.

Further details of the sensor fabrication and performance as LDV are presented in our previous report [20]. The picture of the µ-LDV chip and the chip installed on PCB (printed circuit board) with pre-amplifier circuit are shown in Figure 4a,b, respectively. Although the total size (18 × 16 × 2.5 mm-thick) might be slightly larger than the human fingertip, more than half of the area is occupied by holes for fixing, large wiring space for hand soldering, redundant resistance elements (6 of 10 pieces), and a redundant amplifier channel (one of two channels) for another testing. All elements are mounted on a single-side. Therefore, if double-side mounting structure is adopted, and redundant elements are eliminated, the size can be miniaturized (i.e., one-third in volume and one-sixth in foot-print of the present size or less) with available circuit board manufacturing technology. 

### 2.3. Grasping Model

Many of the slip sensors developed to date—excluding the heat flux type—have elastic surfaces, just like humans have elastic skin on their fingertips for detecting microscopic dynamical physical change induced by slip. On the other hand, with an LDV, slip is contactlessly detected as relative displacement between the sensor surface and target object surface. Thus, friction conditions at the measurement point are irrelevant to the slip sensing of a target object. The contactless measurement is one of distinctive features of LDV type slip sensors. In setting up a grasping model, many factors must be considered such as contact points, number of gripper fingers, type of actuators, deformation of gripper and target objects, gripper kinematics and dynamics, gravitational and translational acceleration forces, and sensory feedback; and accordingly, a variety of grasping models have been proposed as discussed in references [21,22,23]. Larger contact surfaces and a higher number of gripper fingers improve the grasping stability, although they make the model large and complex. Various types of mechanical grippers have been proposed. There are many choices of gear types used as kinematic for grippers: lever gear, screw gear, wedge gear, cam gear, rope and pulley, link, and rack and pinion gear [23,24,25,26]. The increasing degree of freedom of a robotic hand by possessing a lot of gears, links, and intelligent algorithm, a robotic hand can achieve dexterous manipulation similar to human—for example, optimization of its orientation, surface shape, and size to a target object for grasping—although more complex control algorithms and force analysis are necessary [27,28,29,30,31,32,33,34,35]. There are also many choices of actuators such as mechanical, electric motor, pneumatic, hydraulic, and magnetic [36,37,38,39]. Actuators must be chosen carefully by considering factors such as controllability, size, required grasping force, and so on [23]. 

**Figure 5:**
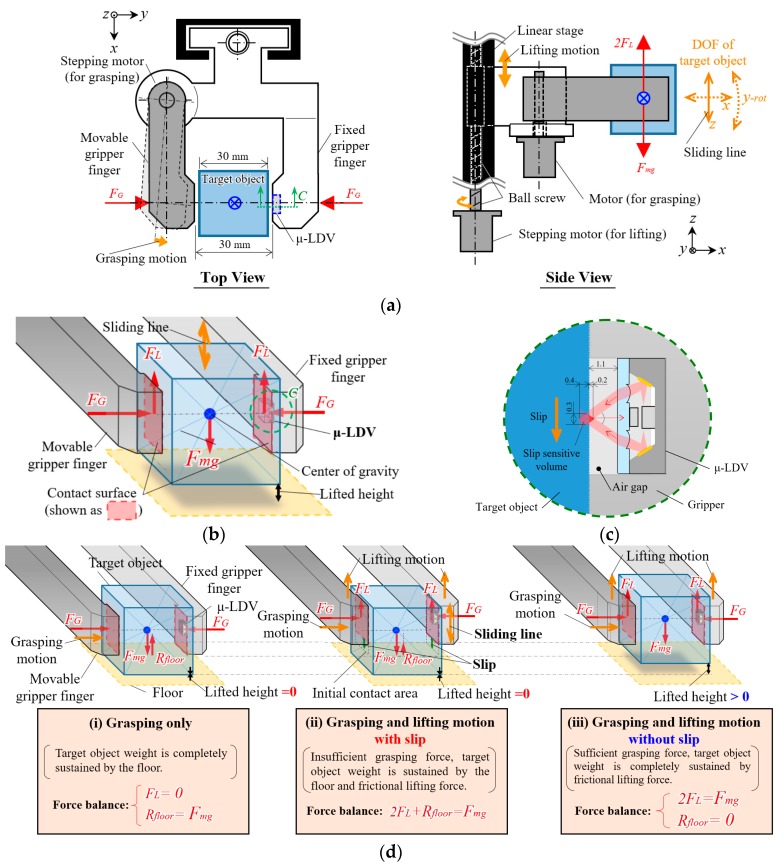
Grasping model: (**a**) Mechanical model of grasping. (**b**) Schematics of description of contact surfaces and force balance in grasped condition. (**c**) Description around slip detection sight (“C” shown in (**a**,**b**)). (**d**) Force balance, sliding line, and direction of grasping motion and lifting motion around a target object in the model in (**i**) only grasping, (**ii**) grasping and lifting motion with slip, and (**iii**) grasping and lifting motion without slip.

Considering the aforementioned features of LDV and factors involved in the model of grasping, we built the grasping model for the μ-LDV testing as shown in Figure 5. At least two actuators are necessary for the grasping test: grasping actuator and lifting actuator. We chose a stepping motor as the actuator for both grasping motion and lifting motion because of its high controllability. Although the generative force is relatively smaller than other types of actuators, the stepping motor can generate enough force with capable size for testing. One of the stepping motors is directly attached to the movable gripper finger. The model pinches a target object with the movable gripper finger and fixed gripper finger by rotating the movable gripper finger around the *z*-axis. The grasping force can be controlled by actuating the stepping motor. We select this grasping construction to be able to perform the grasping test with the simplest testing setup, while many of practically-used grasping models have mechanical links to comply with various shapes of target objects. The grasping model assumes surface contact as described in Figure 5b, since surface contact is more preferable than line or point contacts [23] (in Section 2.2). The surface contact condition cannot be achieved except in conditions where both sides of gripper surface and target object surfaces are parallel to each other at the contact position. Thus, we use a cube 30 mm on each side as a target object, the length of which are the distance between two gripper surfaces when the surface of a movable gripper finger is in a position parallel to the surface of fixed gripper finger. The other stepping motor is attached to the ball screw equipped on the linear stage for lifting motion. Rotary motion of the other stepping motor is converted to linear motion via ball screw, thus, the lifting height and lifting velocity can be controlled by the stepping motor. When grasping force is insufficient, this model, grasping an object with parallel contact surfaces, allows the target object slip along three degrees of freedom (DOF) of axes (*z*-axis, *x*-axis, and around *y*-axis) as indicated in Figure 5a, side view [23] (in Section 2.1). However, we exclude the possibility of slip along *x*-axis and around the *y*-axis, assuming that any external force is never applied to the object, and the gripper grasp at the center of gravity of the object. 

The lifting force is generated on the contact surface as a result of friction. Slip occurs when lift force is insufficient to overcome the gravitational force. In order to prevent slip, grasping force should be increased to give higher frictional force, within the range without causing damage to the target object. Let us estimate the minimum grasping force F_Gmin_ according to the Coulomb’s friction model described in the Introduction,

(2) $$F_{G_{\min}}=\frac{\mathrm{mg}}{2\mu }$$

where m, g, and µ represent weight of a target object, gravity acceleration, and static friction coefficient, respectively. It is known that friction arises against not only the normal force but also the torque force [40]. To exclude torque force in the simplification of the model, the target object is placed so that its center and the center of contact surface are in line. Figure 5c shows the dimensional description around the slip sensor. The µ-LDV is embedded into the cavity of a gripper finger, the depth of which is designed so that the cross point of laser beams locates near the gripper surface plane. Therefore, there is an air gap between the surfaces of the µ-LDV and the target object, and slip is measured without contact. The laser cross volume is the slip sensitive area, and the distance from gripper surface plane is from 0.2 mm inner side to 0.4 mm outer side. This long sensitive area gives the sensor robustness of slip detection against non-smooth surface. Figure 5d explains force balance, sliding line, and direction of grasping motion and lifting motion around a target object in the model in (i) only grasping, (ii) grasping and lifting motion with slip, and (iii) grasping and lifting motion without slip. In condition (i), target object is grasped but lifting force F_L_ is zero since the gripper is not lifted up, thus, still no friction is generated. Target object weight is completely sustained by the floor reaction force R_floor_, hence, R_floor_ = F_mg_. In condition (ii), lifting force F_L_ arises during the lifting motion; however, the object cannot be lifted up, then relative slip occurs along the z-axis (gravity direction) due to insufficient grasping force. Forces are balanced as 2F_L_ + R_floor_ = F_mg_. In the condition (iii), the object is successfully lifted up. Lifting force and gravitational force are balanced as 2F_L_ = F_mg_, and of course, floor reaction force is zero. The forces are kept balanced while grasping force over the border is enough to generate frictional force against the weight of the object. 

### 2.4. Experimental Setup

**Figure 6:**
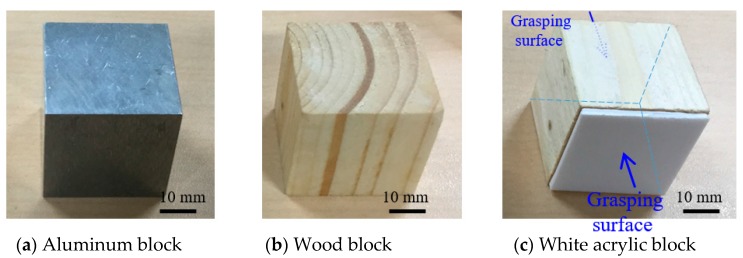
Pictures of target objects for grasping. (**a**) Aluminum block; (**b**) Wood block; (**c**) White acrylic block.

We used three types of blocks as target objects: aluminum block, wood block, and white acrylic block (Figure 6). We have selected these three testing materials from one of each representative material categories that can be found in homes such as metal, wood, and resin, assuming slip detection by a slip sensor for home-assist robots. The size, weight, static friction coefficient, and estimated minimum grasping force of the target objects for grasping are listed in Table 1. 

**Figure 7:**
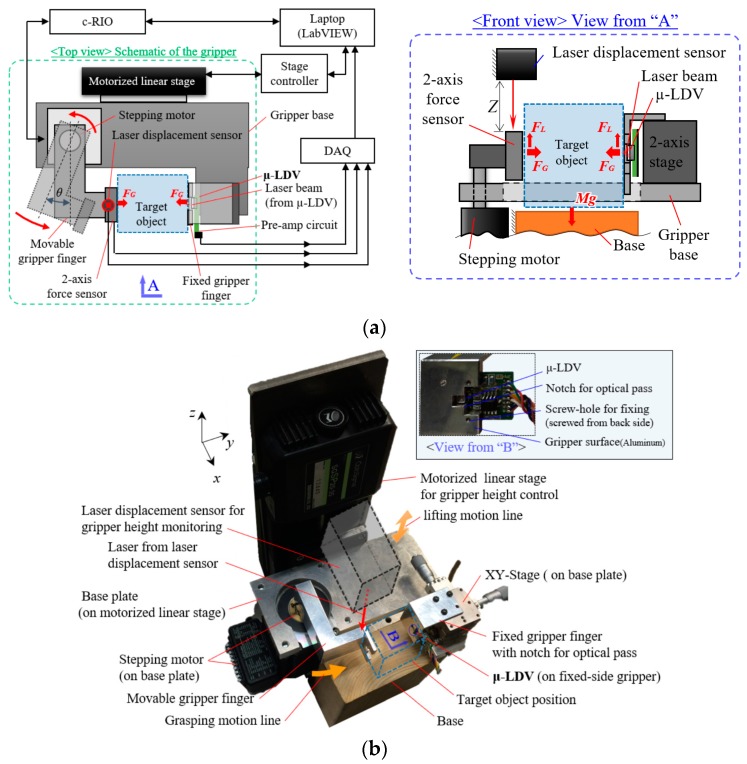
Experimental setup: (**a**) A schematic of experimental setup. Grasping motion is controlled by a stepping motor, operated by LabVIEW via c-RIO interface. Gripper height is controlled by a motorized linear stage, operated by LabVIEW via stage controller interface. Output signals of the μ-LDV, two-axis force sensor, and a laser displacement sensor for gripper height monitoring are processed and recorded by LabVIEW via DAQ interface. The figure at the right side shows the front view (from the view point “A”), with the relation of force vectors on target object. Motorized linear stage is omitted; (**b**) a picture of gripper setup. A laser displacement sensor and a target object are additionally illustrated. The μ-LDV is fixed on L-shaped gripper with a notch for optical path. Inset shows description around the μ-LDV from the view point “B”.

We built the experimental setup according to the grasping model defined in Section 2.3. Figure 7 explains the experimental setup: (a) Schematic of the experimental setup, and (b) a picture of the gripper setup. Grasping motion was controlled by rotating a stepping motor, operated by LabVIEW (National Instruments Japan Corp., Tokyo, Japan) via compactRIO interface (programable controller interface, cRIO-9063 and NI9512, National Instruments Japan Corp., Tokyo, Japan). Gripper height was controlled by a motorized linear stage (SGSP20-35, Sigmakoki Co., LTD. Tokyo, Japan), operated by LabVIEW via stage controller interface. A laser displacement sensor (LK-G15, Keyence Corp., Osaka, Japan) monitored the gripper height. Output signals of the µ-LDV and two-axis force sensor (USL06-H5-50N, with signal amplifier DSA-03A, Tech Gihan Co., Ltd., Kyoto, Japan) were processed and recorded by LabVIEW via compactDAQ interface (Data Acquisition interface, cDAQ-9174 and NI9215, National Instruments Japan Corp., Tokyo, Japan). The right-side of Figure 7a shows the front view of the setup (from the view point “A”), with the relation of force vectors to the target object. The μ-LDV was fixed on an L-shaped gripper with a notch for optical path as shown in the inset of Figure 7b.

### 2.5. Signal Test and Threshold Determination of Slip Descrimination

**Figure 8:**
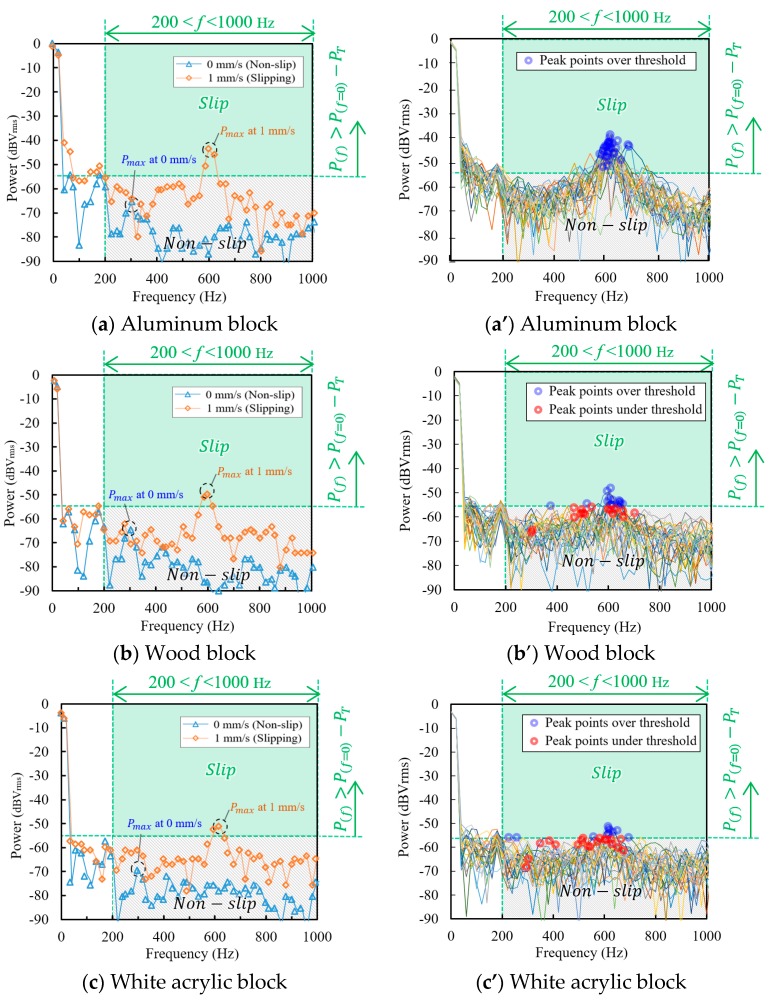
Examples of power spectra of the µ-LDV output in non-slip and slipping at 1 mm/s. The green-colored area indicates the region that fulfills slip determination condition. The control system judges slipping when P_max(200<f<1000)_ is in the green-colored area: (**a**) Aluminum block; (**b**) Wood block; (**c**) White acrylic block. (**a’**–**c’**) show 30 continuous power spectra of each block measurement during slipping. The P_max(200<f<1000)_ of each power spectrum is indicated as a blue circle if the power is higher than P_(f=0)_ − P_T_ (Equation (3) is ‘true’), or as a red circle if the power is lower than P_(f=0)_ − P_T_ (Equation (3) is ‘false’).

Before the grasping force control test, we performed a pre-test of the µ-LDV with the experimental setup, to find the slip determination condition. Each block was placed just in front of the fixed gripper finger surface with slight grasping force to ensure that slip occurs. Then, the motorized stage lifted the gripper to a 1.5 mm higher position at a velocity of 1 mm/s to create a slip state. The power spectra of the µ-LDV output voltage for each target blocks during stop and slipping at slip velocity 1 mm/s was recorded in LabVIEW. Fast Fourier transform (FFT) condition for power spectrum calculation is listed in Table 2. The partial data of pre-test results are shown in Figure 8a–c, respectively. As for the result from aluminum block (Figure 8a), the shape of the two power spectra were different from each other in the frequency range from 200 Hz to 1000 Hz. We named the maximum power in the frequency range as P_max(200<f<1000)_. The power spectrum at slipping has a peak at 600 Hz, the peak frequency corresponds to theoretical frequency of the µ-LDV output for 1 mm/s as expressed in Equation (1), while the power spectrum at non-slip has no conspicuous peak but noise floor, with a power around −80 dBVrms. Thus, slip can be judged by distinguishing the difference between these power spectra. The power spectra at slipping in wood block (Figure 8b) and white acrylic block (Figure 8c) also showed maximum peaks around 600 Hz similar to that of the aluminum block, except that the peak powers are smaller. However, when we referred to all power spectra during slipping, we found that some of the P_max(200<f<1000)_ were lower compared with those of the aluminum block. The difference could affect the sensitivity of slip detection. Comparing those power spectra, we determined slip determination condition on the basis of P_(f=0)_ as

P_max(200<f<1000)_ > P_(f=0)_ − PT (3)

where, P_(f)_ is the power at frequency f and thus, P_(f=0)_ means DC power of the output, P_T_ is −53 dBVrms, a constant to define the threshold. When Equation (3) is “true”, the experimental setup discriminates the target object as slipping. We applied DC power value to the determination equation, aiming to reduce the influence of optical property difference (scattering, transmission, absorption, and reflection) of target object surface, and slight fluctuation of output laser power output, which might be induced by insufficient temperature control in practical environments. “true” area is also graphically indicated as green-colored area in Figure 8. Let us now consider the stability of slip detection. Figure 8a’–c**’** show all power spectra that were continuously obtained during slipping, that is 1.5 mm vertical travel distance at 1.0 mm/s velocity. Since the total travel time is 1.5 s and FFT period is 50 ms, 30 power spectra were obtained from each block. The P_max(200<f<1000)_ of each power spectrum is indicated as a blue circle if the power is higher than P_(f=0)_ − P_T_ (Equation (3) is “true”), or as a red circle if the power is lower than P_(f=0)_ − P_T_ (Equation (3) is “false”). As for the (a’) aluminum block, the power spectra were stable. All P_max(200<f<1000_ of each power spectrum had peak around 615 Hz, and powers of which were over the threshold P_(f=0)_ − P_T_. On the other hand, some of the P_max(200<f<1000)_ of power spectra of (b’) wood block and (c’) white acrylic block were not over the threshold, resulting to loss of slip detection. In order to increase the reliability of slip detection, we introduced the “true-rate” as discussed later in Section 2.6, which is the percentage of “true” signals during lifting motion.

### 2.6. Control Scheme of Grasping Force Control

The control system must be considered from the point of view of four functions: manipulation ability, sensory ability, data processing ability, and intelligence ability that is the capability of exploiting information to modify system in a pre-programmed manner [21]. As described in Section 2.4, the experimental setup has two actuators; a uniaxial motorized stage for lifting motion, and a stepping motor for grasping motion. Each of actuator position can be ordered by LabVIEW via each interface in sufficient control accuracy of 1 µm for lifting motion, and 1/25,000 rotary resolution by feeding back each embedded encoder signals to each interface. The performance of the µ-LDV was confirmed in Section 2.5. The µ-LDV outputs analog signal continuously, then it is FFT-analyzed, and determines the target object to be slipping or not, along with Equation (3) by LabVIEW, every 50 ms. The LabVIEW judges whether it is increased grasping force or end grasping force control from the slip discrimination results during the lifting motion. Lifting motion and slip discrimination are continued until slip is no longer observed.

**Figure 9:**
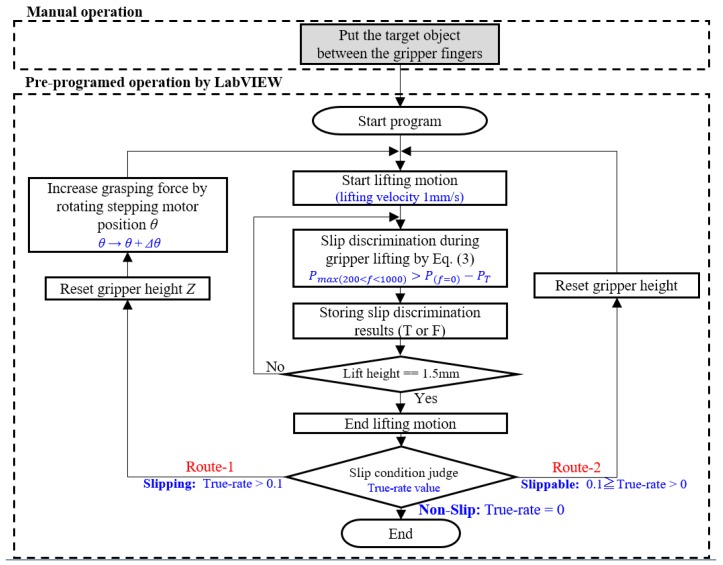
Sequence of grasping force control with the μ-LDV signal.

The sequence of grasping force control with the µ-LDV is shown in Figure 9. At first, we manually put a target object between the grippers, touching to the target surface with a very low contact force. Next, we start grasping force control test program in LabVIEW, then, motorized stage lifts gripper from initial height to 1.5 mm higher position at lifting velocity 1 mm/s. Output signals of the µ-LDV is recorded and FFT-analyzed in real-time. Power spectrum is sent and determined to be slipping or not as soon as they are obtained, and the discrimination results are stored in the memory in LabVIEW. This signal processing is repeatedly processed in every 50 ms during the gripper lifting. We embedded the true-rate parameter in the control sequence to make the slip discrimination stable as defined in Section 2.5. If the true-rate is more than 0.1, the test continues along with “Route-1”: reset the gripper height to the initial position, and then, increase the grasping force by rotating the stepping motor in fixed rotation steps, 5, that is equal to 360 × 5/25,000 degrees. After that, gripper lifting and slip determination process are restarted. In the case of the true-rate which is from 0 to 0.1 at the slip condition judge process, the test continues along with “Route-2”: reset the gripper height to the initial position, and then restart gripper lifting without increasing grasping force. The sequence will be continued until the true-rate becomes 0. The applied force onto the gripper surface is continuously monitored by the two-axis force sensor during the test. 

## 3. Results and Discussion

**Figure 10:**
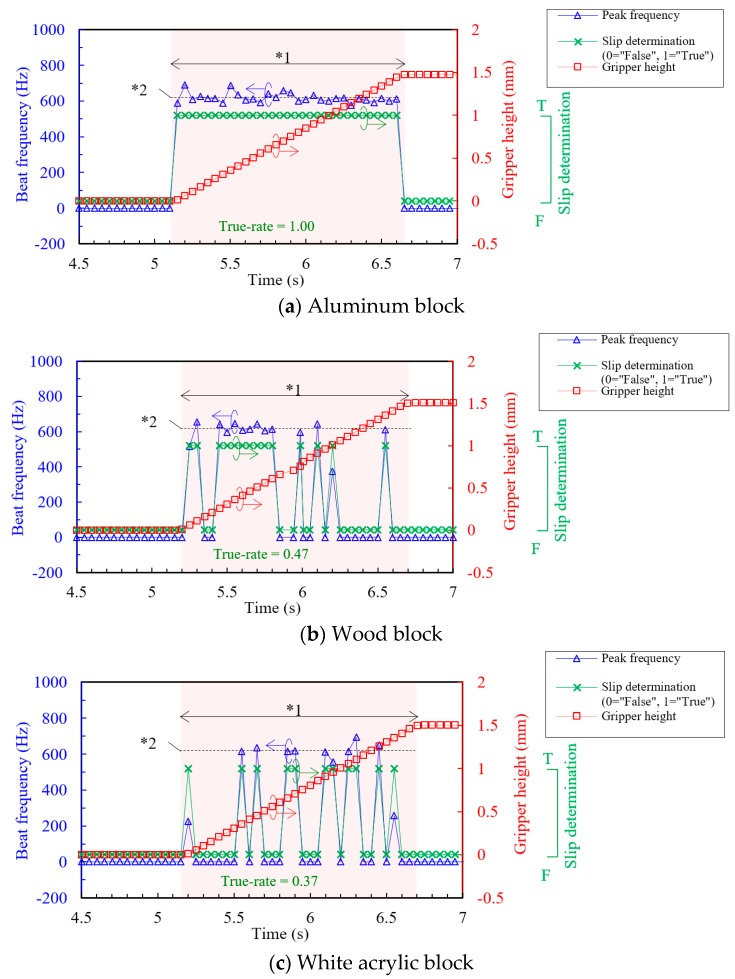
Examples of power spectra of the µ-LDV output in non-slip and slipping condition at 1 mm/s slipping velocity. The green color indicates area of fulfilling slip determination condition: (**a**) Aluminum block; (**b**) Wood block; (**c**) White acrylic block. *^1^ Duration of gripper lifting at 1 mm/s. *^2^ Theoretical beat frequency at 1 mm/s, calculated by Equation (1).

Figure 10a shows the result of the aluminum block target at first gripper lifting, when the gripper applied small grasping force that is obviously not enough to lift up the block. The orange-colored area indicates the duration of the gripper lifting at the velocity 1 mm/s. The results of slip determination were plotted as 1 for “true” (slipping) and 0 for “false” (non-slip). The slip determination result showed “true” during lifting, and “false” during stop, perfectly. The beat frequency, which is the frequency at power peak of each power spectrum, was also plotted. The beat frequency is linearly related to the slipping velocity, thus it could be an optional parameter for more efficient grasping control algorithm, although it was not feedbacked to the grasping control in this test. The theoretical beat frequency for slip velocity 1 mm/s is 615 Hz, thus the μ-LDV was correctly detecting the slip velocity of the aluminum block. This result means that the µ-LDV can determine slip condition and measure slip velocity in real time in high response speed, 50 ms.

As for the results at the first gripper lifting for the wood block (Figure 10b) and the white acrylic block (Figure 10c), slip determination result randomly output “true” and “false” during the gripper lifting. On the other hand, it output only “false” when the gripper stopped. As aforementioned, we introduced true-rate to make the grasping control process reliable. T-rate at the first cycle were 1.00 for the aluminum block, 0.47 for the wood block, and 0.37 for the white acrylic block. As all the values were more than 0.1, grasping force control program judged that target objects were slipping, and then, the program continued in accordance with “Route-1”: reset the gripper height and increased grasping force. After that, the second gripper lifting process started. 

**Figure 11:**
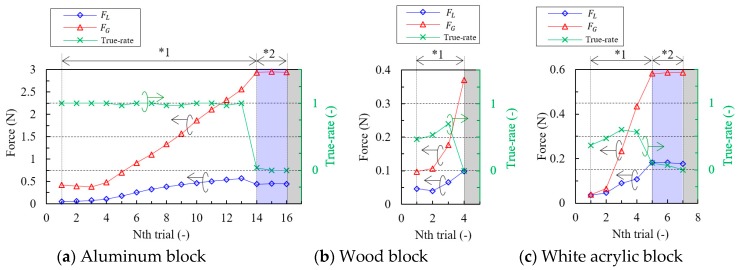
Forces and True-rate at each gripper lifting: (**a**) Aluminum block; (**b**) Wood block; (**c**) White acrylic block. Note that vertical scales are different for each block. *^1^ Processed via ‘Route-1’ and *^2^ Processed via ‘Route-2’ as explained in Section 2.6, Figure 9.

Figure 11 shows the complete result of grasping force control test. Grasping force, lifting force, and true-rate for each lifting trial are plotted. As for the result of aluminum block (Figure 11a), at the beginning of the trial, the true-rate was 1 or slightly smaller than 1, meaning the block was completely slipping. Since stepping motor was rotated to increase the grasping force when the true-rate was over 0.1 (Route-1), grasping force and lifting force were gradually increased as the cycle of the trial progressed. At the fourteenth trial, true-rate suddenly decreased, and the resulting grasping of the block was almost accomplished. However, it should be noted that slip occurred slightly. Thus, we applied the other sequence for the slight slip observed, called “Route-2” as aforementioned in Section 2.6, and in Figure 9. This sequence works as re-grasping process without changing grasping force. After two trials of Route-2 sequence, the true-rate became exactly 0, then grasping force control test ended. The final grasping force and lifting force were, 2.94 N and 0.44 N, respectively. 

Regarding the result of wood block (Figure 11b) and white acrylic block (Figure 11c), True-rate during slipping was from 0.37 to 0.7, which is roughly half compared to that of aluminum block. The number of trials until grasping control sequence ends, and final grasping force and lifting force were, 4, 0.37 N, 0.10 N for wood block, and 7, 0.59 N, 0.18 N for white acrylic block, respectively. We confirmed that the target objects were grasped and not slipping. Note that grasping control sequence, slip determination condition, FFT condition, and any other conditions and setup were fixed in all the experiments. 

To compare the results and estimated minimum grasping force by Coulomb’s friction equation in static friction and dynamic friction, we summarized them in Table 3. The experimental result was 4.2 to 5.3 times higher than the estimated value in static friction and from 3.3 to 3.4 times higher than the estimated value in dynamic friction. Although the experimental results are higher than the estimated value, target objects were slipping when the grasping force was lower than the final grasping force for each experiment, thus the final grasping force could be considered as near-minimum grasping force for the experimental setup. To obtain a final grasping force closer to the estimated value, the following measures are considered: (i) Set smaller incremental step of grasping force for higher resolution of force control; (ii) Substitute actuators with ones that move more smoothly since the present actuators create micro-vibrations which are considered to be the cause for target objects to slip easily; (iii) Increase flexibility of grippers to avoid edge contact that may occur during the grasping test, to make contacting condition stable; (iv) Reconsider estimation model to obtain closer value to the true minimum grasping force. Several papers discussed the minimum required grasping force by using the Coulomb friction equation (references [1,9,23]), and thus, we consider the theory to be effective at least for approximate estimation of grasping force. However, the estimation model may not be enough to discuss true minimum grasping force because the actual friction coefficient depends on many factors—such as contaminants like moisture, oil, and dust—and it may vary even for the same material [41]. In addition, we technically depict the actual friction to possess the dynamic characteristic described as Stribeck friction model [42,43].

Regarding the response speed, the μ-LDV is capable of real-time slip detection for the aluminum block. As for the wood block and the white acrylic block, however, response speed is not very high since detection loss of slip was randomly observed, and there was a need for the time to process several times of FFT to obtain the true-rate. To improve the response speed, slip detection loss must be reduced. Considering the reason why the detection loss occurs, we focused on three points: (i) the slip condition was in stick-slip; (ii) low S/N (signal–noise) ratio; and (iii) optimizing slip determination condition. Although the slip velocity must fluctuate if the slip condition is in stick-slip, beat frequencies for the wood block and the white acrylic block did not obviously fluctuate compared with the aluminum block (Figure 10). Thus, it is considered that slip condition was not in stick-slip. The S/N ratios of the power spectra from wood block and white acrylic block were lower than aluminum, resulting in the impossibility of determination of threshold setting. It is needed to achieve high response speed for grasping various target surfaces to improve the S/N rate, for example, by increasing the laser intensity, improving the PD sensitivity, improving coherence of laser beams, and performing noise countermeasures for the signal processing circuit. In the test, slip determination condition was determined with only peak power and its frequency range. Thus, it is necessary to consider a more effective determination condition, such as using the shape of power spectrum and applying DWT (discrete wavelet transform) [8,44] because the signal of μ-LDV may possibly have useful information in both the time and frequency domains. 

However, we also looked into the possibility of a situation wherein certain slip detection might be difficult with the μ-LDV. Since an LDV measures velocity by detecting scattering light intensity, sufficient scattering light intensity is necessary for the PD and its amplifier circuit. We performed slip detection signal test with the clear acrylic block with fully polished surface (surface roughness R_q_ is 60 nm), according to the same protocol in Section 2.5. However, the PD could not show any signal change, even when the irradiating laser was turned on and off (data not shown). We also considered another case of measurement using a clear vessel like a PET bottle used as a beverage container that contains some light scattering liquid—e.g., fruit juice. An LDV will erroneously detect slip when beverage moves in the bottle, even if the bottle is securely grasped. To eliminate the concern of misdetection, higher spatial resolution for having sensitivity against the bottle surface, and having insensitivity against beverage motion are essential. Further improvement of both the S/N ratio and spatial resolution may be necessary to achieve sufficient slip sensitivity for utility as a universal slip sensor against any object in homes and other practical environment. These are still challenging issues for other types of slip sensors as well.

## 4. Conclusions

Various slip detection devices and methods have been developed. However, the application of laser Doppler velocimeter as a slip sensor for a robotic hand fingertip has been limited due to its large size. We developed a few-millimeter-scale LDV (μ-LDV) which could be embedded into a robotic fingertip. By using the μ-LDV, we successfully performed grasping force control with the consistent algorithm, without feedbacking grasping force for the three types of target objects—aluminum block, wood block, and white acrylic block—as target objects for grasping which are of different weights, friction coefficients, and optical properties. In the measurement principle, laser Doppler velocimeter can measure slip velocity of anything if it scatters light, the reason why laser Doppler velocimetry has a wide range of practical application. Although there are some points to be improved—such as slip determination condition, S/N ratio of the signal for higher sensitivity, and response speed—we have shown that the feasibility of μ-LDV application as a slip sensor for grasping force, as well as control of various types of targets of unknown weight, surface frictional property, and surface optical property. The advantages of LDV such as high response speed and robustness for measurement target, along with the need for miniaturization, lead to the direction of μ-LDV as a new useful slip sensor for a robotic hand application.

## Figures and Tables

**Table 1:** Specifications of target objects.

Block Name	Size (mm^3^)	Weight (g)	Static Friction Coefficient (-)	Dynamic Friction Coefficient (-)	Estimated Minimum Grasping Force by Coulomb Friction Equation in Static Friction ^1^ (N)	Estimated Minimum Grasping Force by Coulomb Friction Equation in Dynamic Friction ^1^ (N)
Aluminum	30 × 30 × 30	75.6	0.54	0.43	0.69	0.87
Wood	30 × 30 × 30	10.6	0.71	0.48	0.07	0.11
White acrylic	30 × 30 × 30	15.3	0.58	0.42	0.13	0.18

^1^ Calculated by Equation (2).

**Table 2:** Condition of FFT.

FFT Parameters	Value
Sampling rate (Hz)	20,000
No. of sampling (-)	1000
FFT range (Hz)	10,000
No. of frequency division (-)	500
Resolution bandwidth (Hz)	20
Period of FFT (ms)	50
Velocity resolution (µm/s)	32.5

**Table 3:** Result of grasping force control tests and their estimated values.

Block Name	Final Lifting Force (N)	Final Grasping Force (N)	Estimated Minimum Grasping Force by Coulomb Friction Equation in Static Friction ^1^ (N)/Ratio to the Experimetal Result	Estimated Minimum Grasping Force by Coulomb Friction Equation in Dynamic Friction ^1^ (N)/Ratio to the Experimetal Result
Aluminum	0.44	2.94	0.69/4.2	0.87/3.4
Wood	0.10	0.37	0.07/5.3	0.11/3.3
White acrylic	0.18	0.59	0.13/4.5	0.18/3.3

^1^ Calculated by Equation (2).
